# Temporomandibular joint ankylosis—“Knowing when not to operate”: Case report and qualitative systematic review of literature

**DOI:** 10.1002/ccr3.5556

**Published:** 2022-03-06

**Authors:** Kavish Kapoor, Arunkumar Shadamarshan Rengasayee, Rohit Sharma, Nitesh Agrawal

**Affiliations:** ^1^ Department of Radiodiagnosis and Imaging Military Hospital Jalandhar India; ^2^ Wangchuck Lo Dzong Military Hospital Haa Bhutan; ^3^ Command Military Dental Centre (Western Command) Chandi Mandir India; ^4^ Military Hospital Jalandhar Punjab India

**Keywords:** extraarticular TMJ ankylosis, fibrodysplasia ossificans progressiva, myositis ossificans progressiva, TMJ ankylosis

## Abstract

Temporomandibular joint ankyloses (TMJA) may manifest in patients with several predisposing systemic conditions. A case of extraarticular TMJA is presented in a patient diagnosed with fibrodysplasia ossificans progressive (FOP) is presented. The features, diagnosis, and management of TMJA superimposed on this condition are presented through a qualitative systematic review of literature.

## Graphical abstract

Fibrodysplasia ossificans progressive (FOP) is a rare cause of temporomandibular joint ankylosis (TMJA). Temporomandibular joint ankylosis in FOP is an absolute contraindication for surgery since surgery (including routine dental procedures) may lead to recurrence and exacerbation of the condition. Atraumatic dental treatment, preventive dentistry, and genetic counseling must be included in the management protocol.

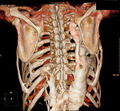

## 1 INTRODUCTION

Temporomandibular joint ankylosis (TMJA) irrespective of the etiology or type leads to a significant compromise in the quality of life in terms of function, nutrition, and aesthetics.^1^ The management of TMJA is essentially surgical.^2^ Early diagnosis, prompt surgery (often multi‐stage), and long‐term follow‐up with a multispecialty team ensure effective comprehensive management of this condition. Several systemic conditions like ankylosing spondylitis and rheumatoid arthritis predispose a patient to developing TMJA and that the management of TMJA in these patients is essentially like those without pre‐existing systemic conditions. We report a case of TMJA as a manifestation of a rare systemic condition, specifically to emphasize one of the most important dictums of medical ethics, “primum non nocere.”

## 2 CASE PRESENTATION

A 60‐year‐old female patient was referred to our center by a general practitioner with pain in her left lower back tooth region of 15 days duration. The diagnosis by the GP was acute apical periodontitis of tooth 38 but only symptomatic treatment could be instituted due to the restricted mouth opening. On elucidation of relevant history, the patient developed restriction in the mouth opening 30 years ago after sustaining trauma to her back after slip and fall‐off stairs. She concurrently started developing gradual restriction in the movement of neck, shoulders, legs, and back leading to difficult daily activities. She experienced intermittent pain in relation to her left lower III molar, which was managed symptomatically over a period of 5 years. She was diagnosed with bilateral knee osteoarthritis and was on conservative symptomatic management for the same; no other contributory medical, surgical, dental, treatment or personal history was evident. She was not a product of a consanguineous marriage, and there was no family history of the disease.

## 3 EXAMINATION

**FIGURE 1:**
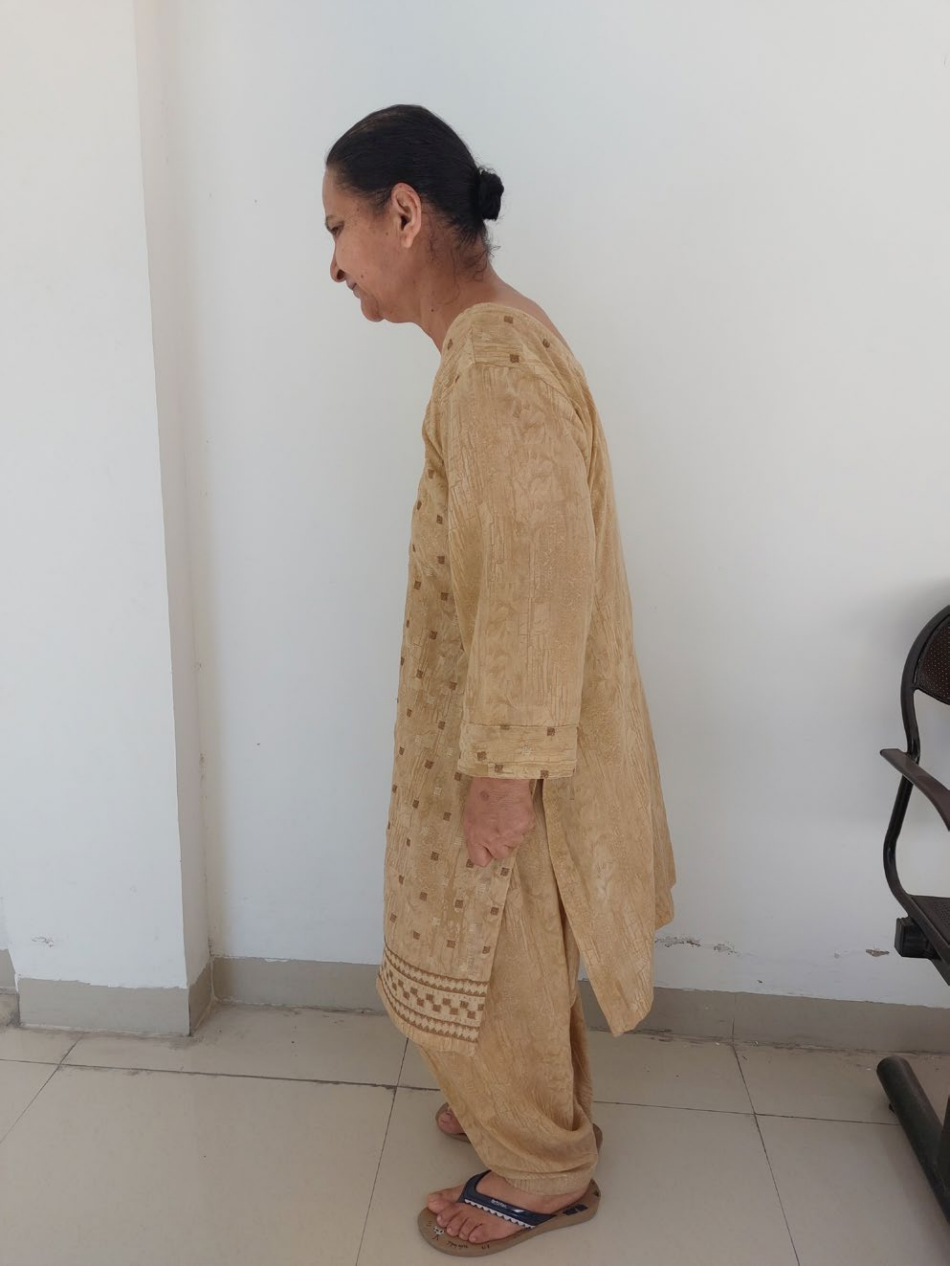
Appearance of the patient

**FIGURE 2:**
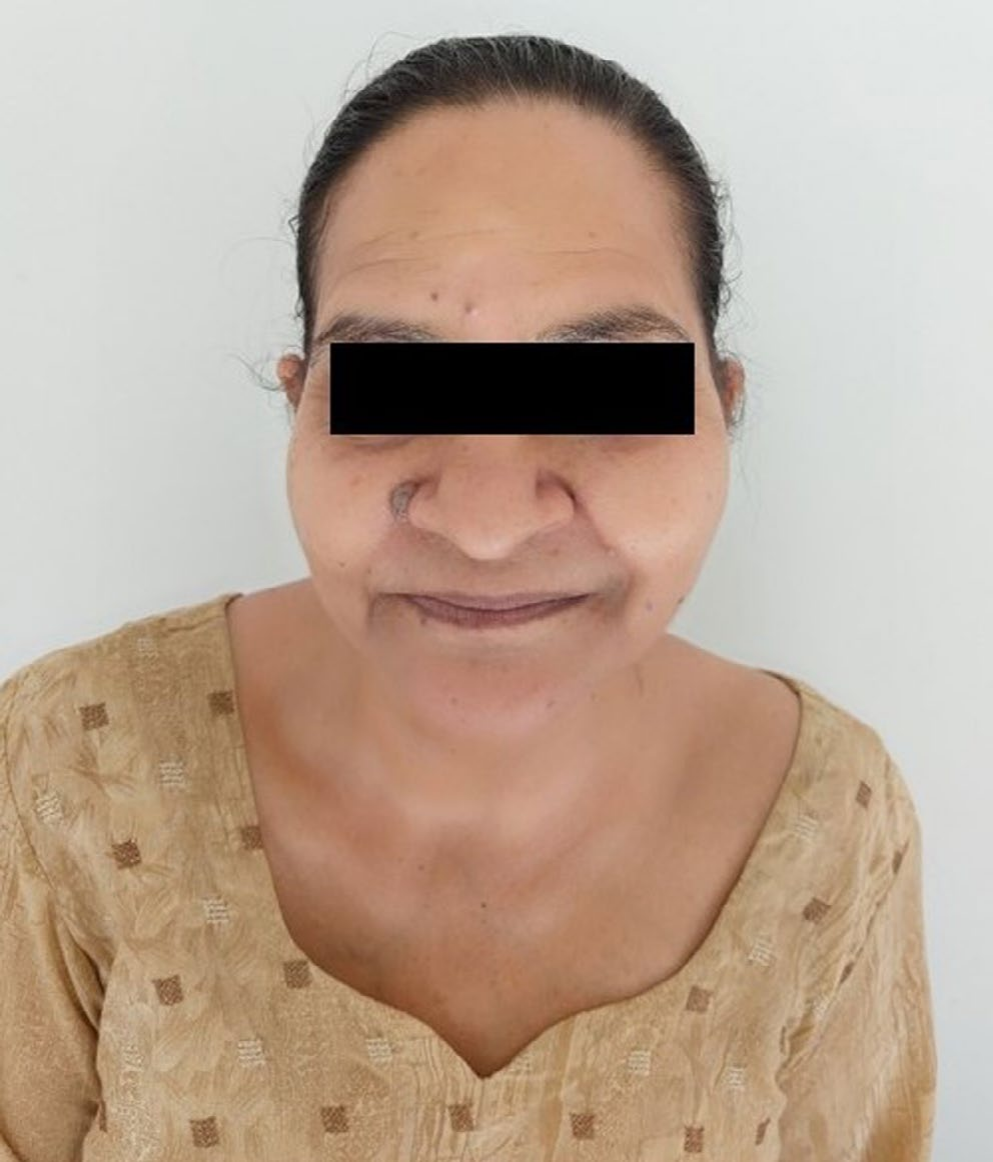
Appearance of the tilted and fixed neck

**FIGURE 3:**
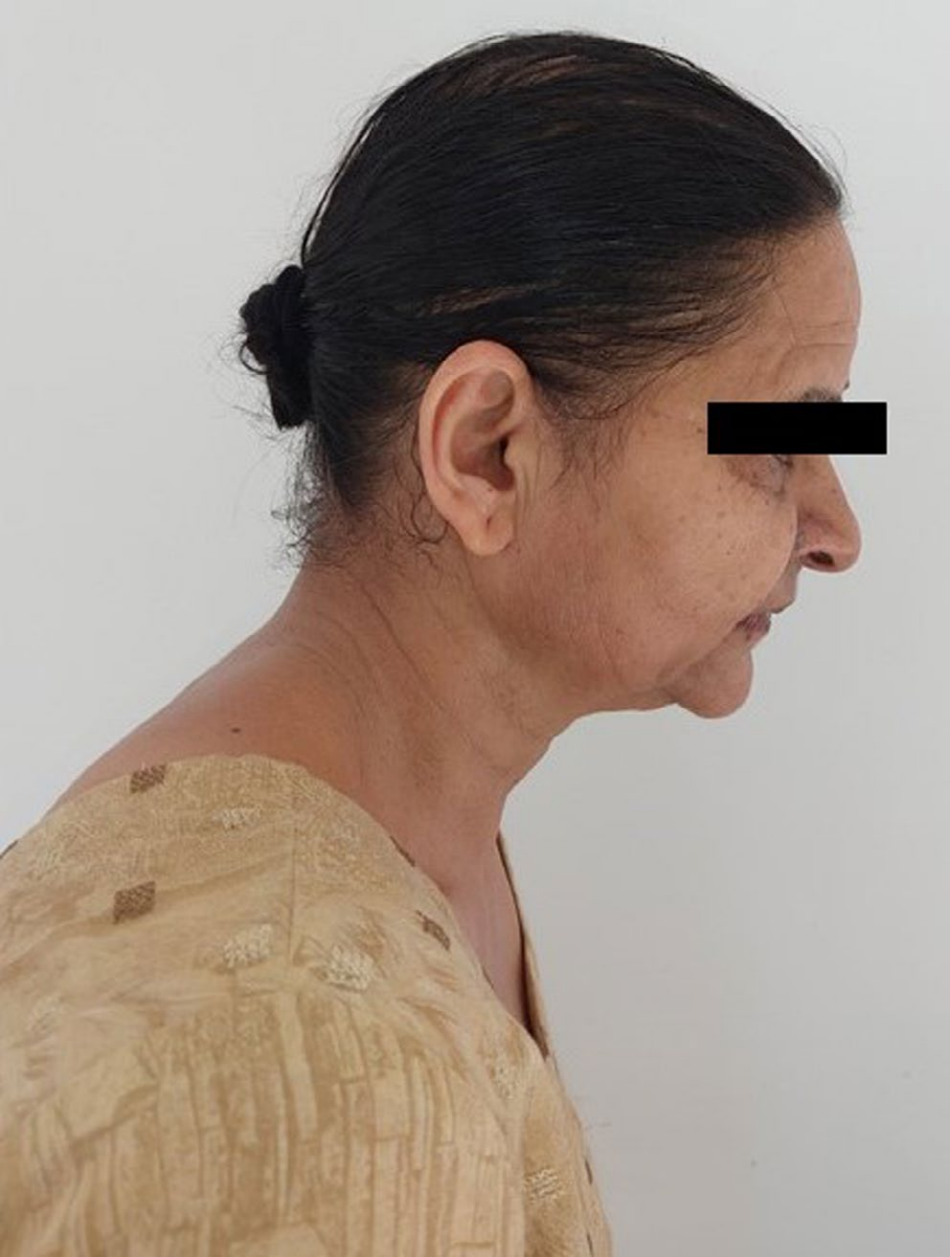
Appearance of the tilted and fixed neck

**FIGURE 4:**
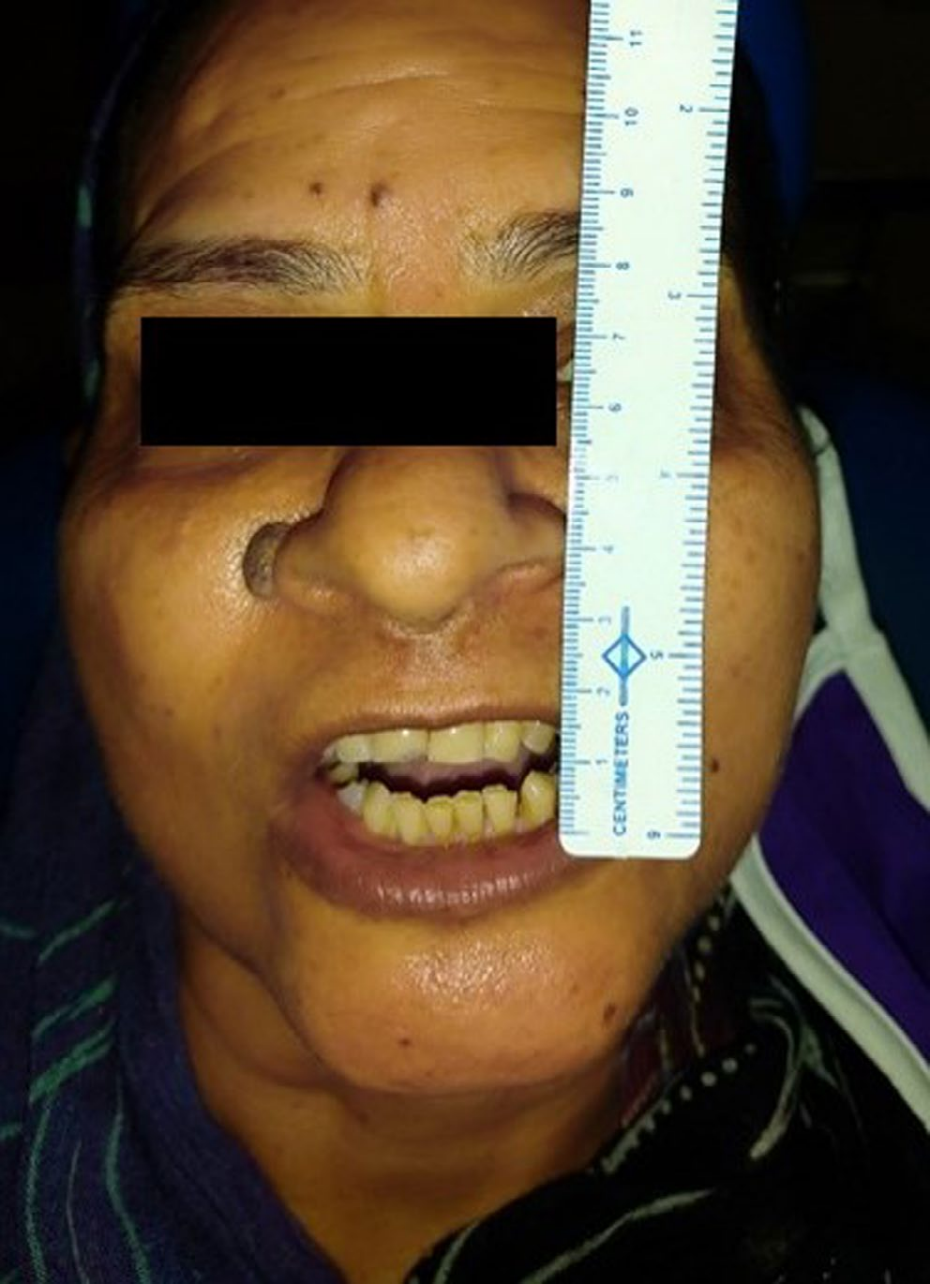
Restricted mouth opening of 4 mm

All systems were normal on routine examination. Vital signs were within normal limits. She had a kypho‐scoliotic appearance with complete restriction in the neck and back movements in all three dimensions (Figure 1). Neck was tilted and fixed to the left side and forward (Figures 2 and 3). Bilateral shoulder movements were restricted. Bilateral hips, knees, and ankles showed normal but painful movement in all three dimensions. On maxillofacial examination, she had a mouth opening of 4 mm with minimal protrusive and laterotrusive movements (Figure 4). The oral hygiene was poor; tooth 38 was grossly decayed and 45 was a decayed root.

## 4 INVESTIGATIONS

**FIGURE 5:**
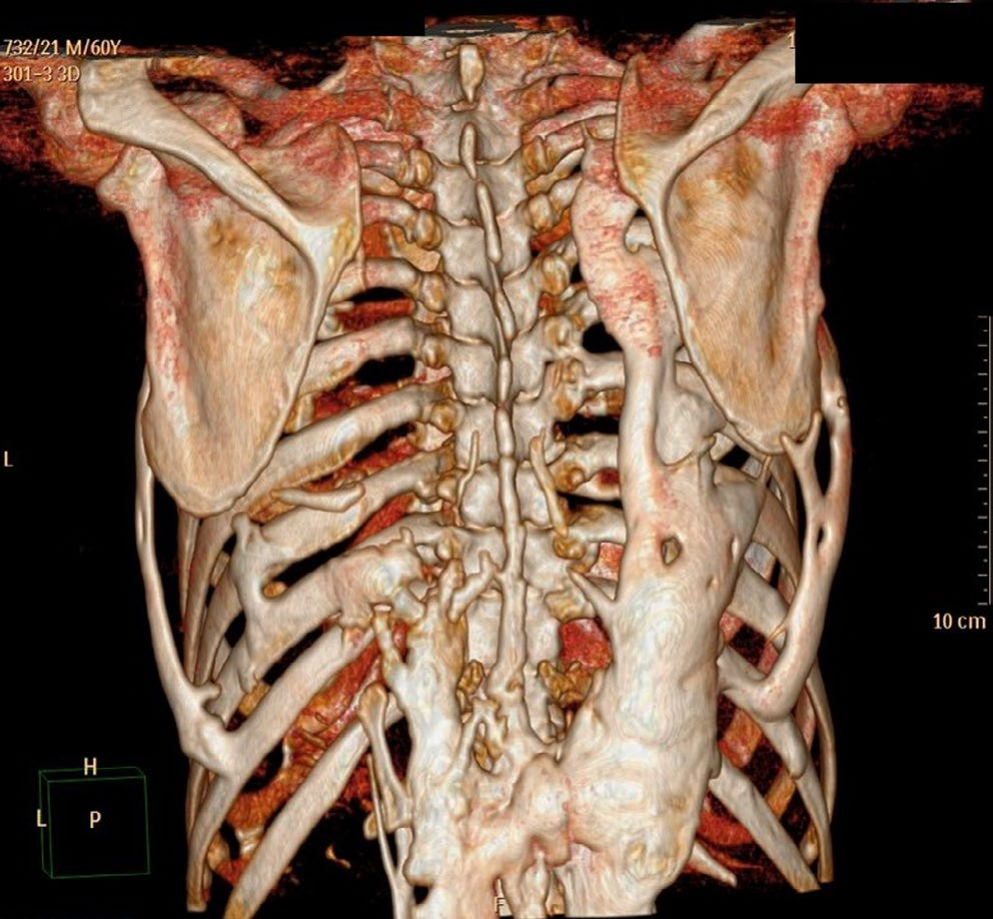
3D Reconstruction of the NCCT chest—Posterior view showing the extent of heterotopic ossification (NCCT—non‐contrast computed tomography)

**FIGURE 6:**
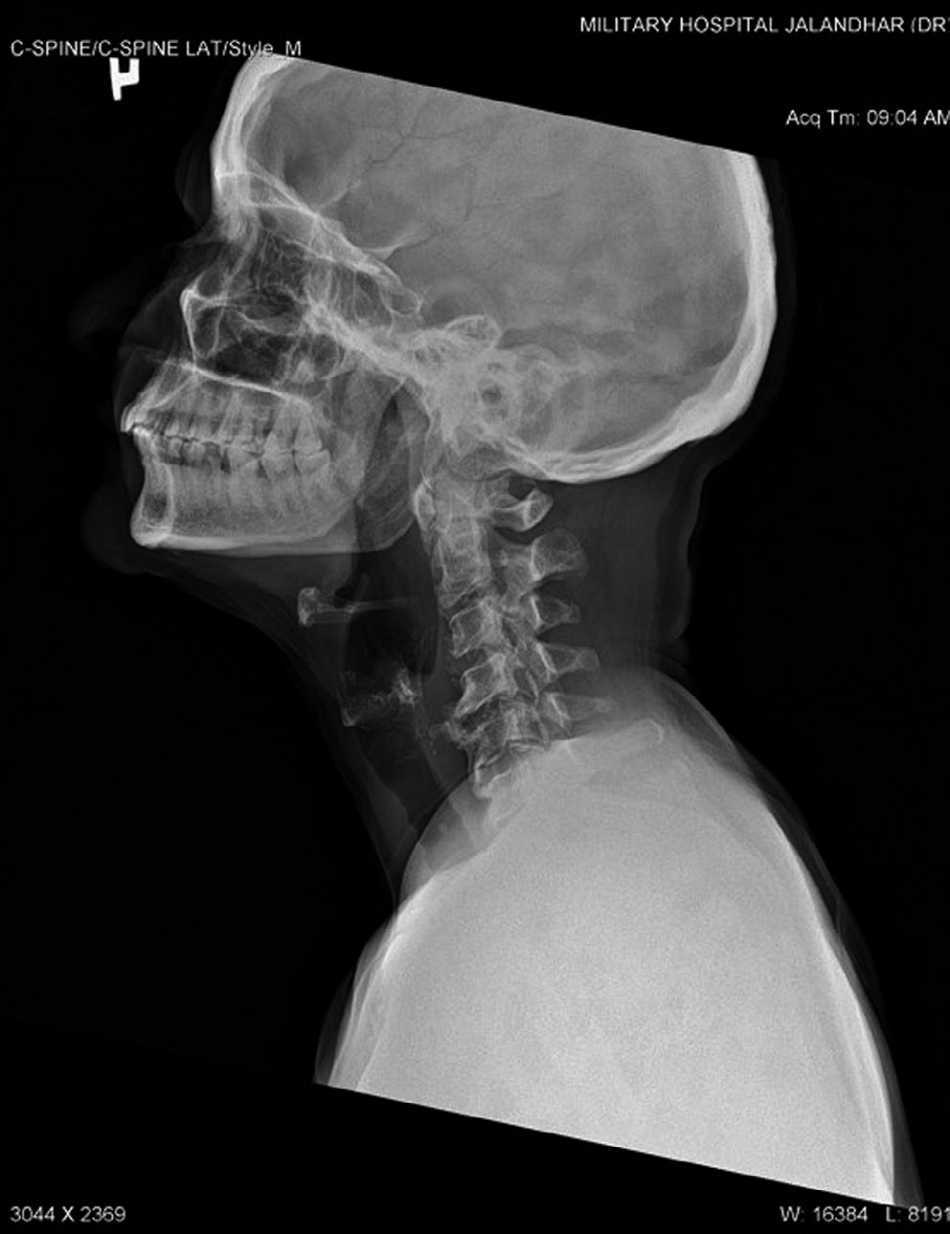
Lateral projection of the neck depicting the fusion of several vertebral bodies

**FIGURE 7:**
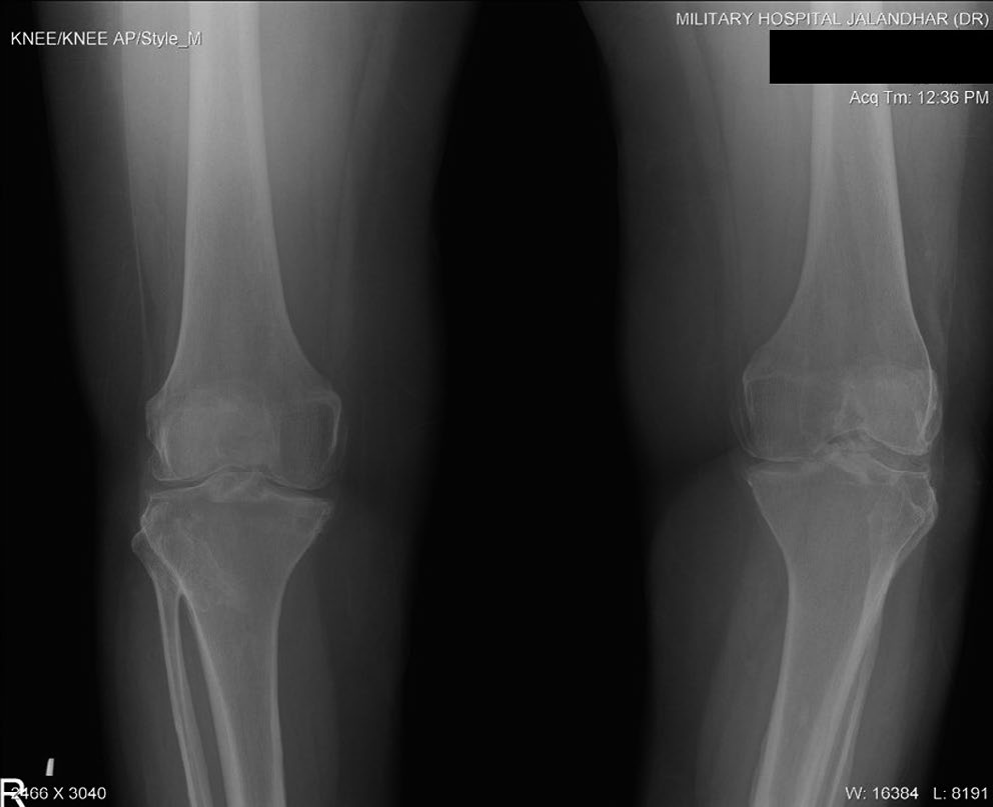
Bilateral Anterior posterior projection of knees

**FIGURE 8:**
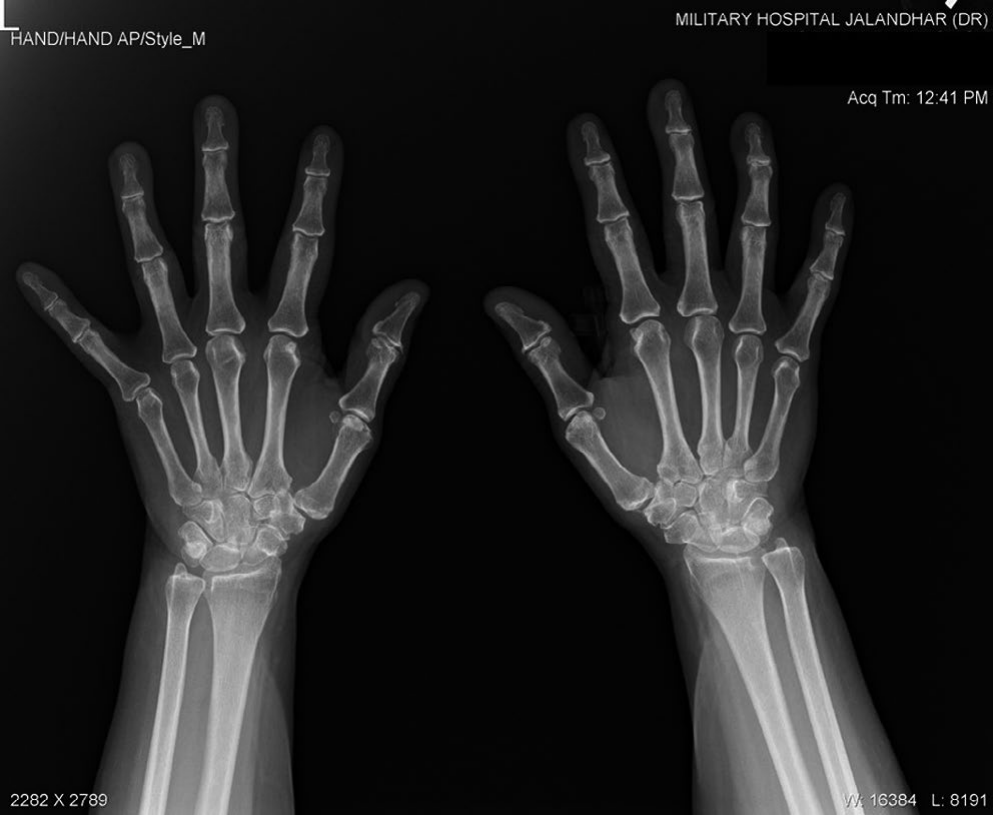
Bilateral hand‐wrist radiographs

**FIGURE 9:**
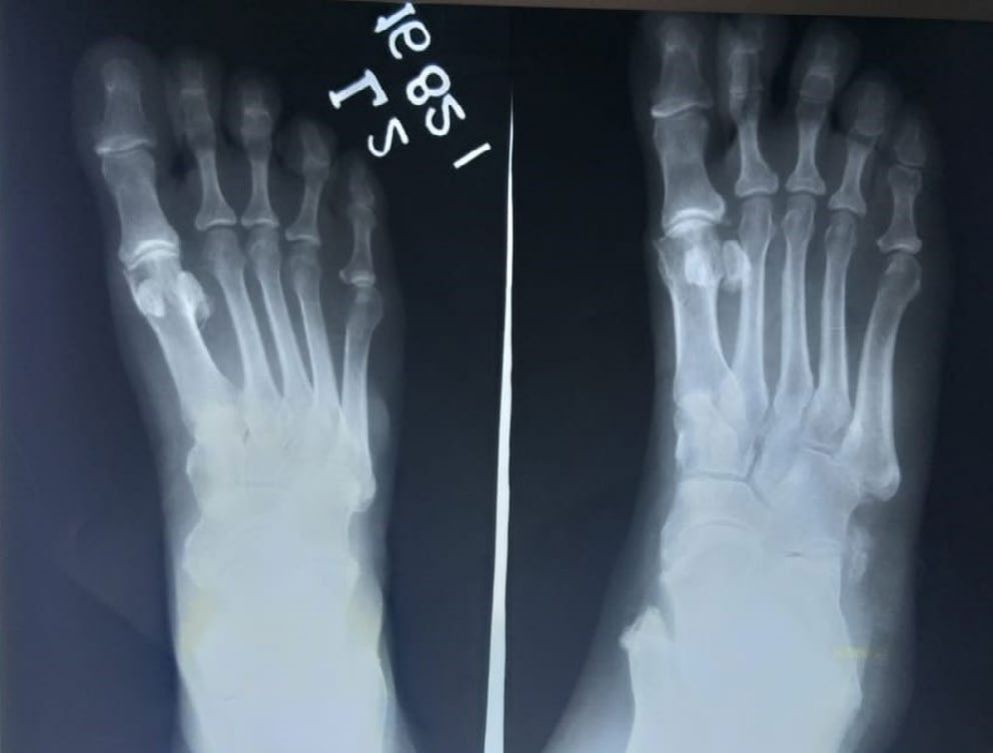
Anterior posterior projection of feet

All routine hematological and serum biochemical investigations were within normal limits, especially erythrocyte sedimentation rate, C‐reactive protein, and serum alkaline phosphatase were unremarkable. Radiographic examination with non‐contrast computed tomography revealed multifocal heterotopic soft tissue ossification and pseudo exostosis predominantly involving the posterolateral aspects of bilateral chest wall (right > left), extending caudally as a plaque‐like ossification in the dorsolumbar region. Synostosis were seen between multiple ribs, vertebral bodies and posterior elements and bilateral scapula (Figures 5 and 6). Degenerative osteoarthritic changes were seen in bilateral knee joints (Figure 7). Hand wrist radiographs were unremarkable (Figure 8). Foot radiographs revealed degenerative changes with reduced first metatarsophalangeal joint with adjacent osteophytic changes (Figure 9). Soft tissue ossification was seen lateral to the cuboid bone and cortical whiskering along the medial malleolus.

**FIGURE 10:**
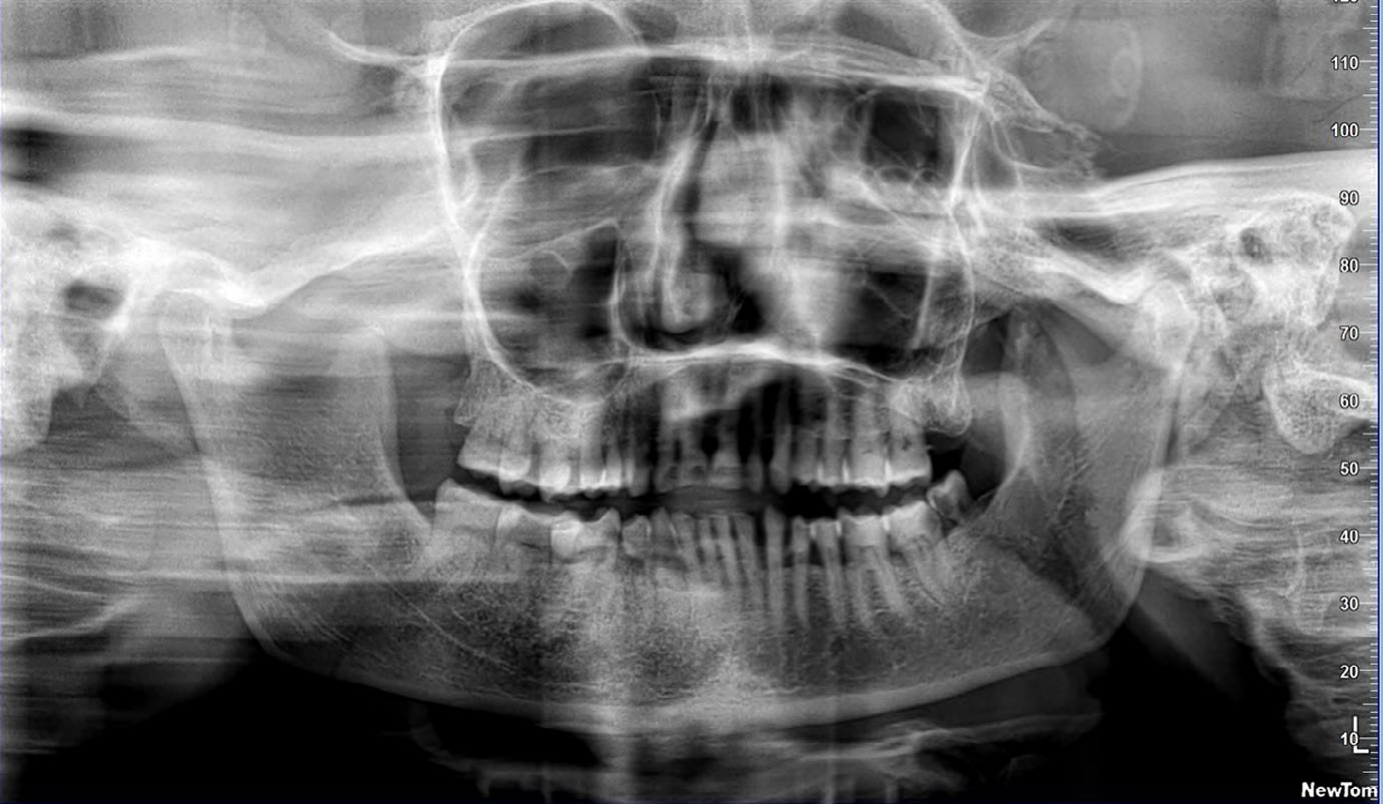
Orthopantogram depicting condylar flattening and bifid tendency on left side

**FIGURE 11:**
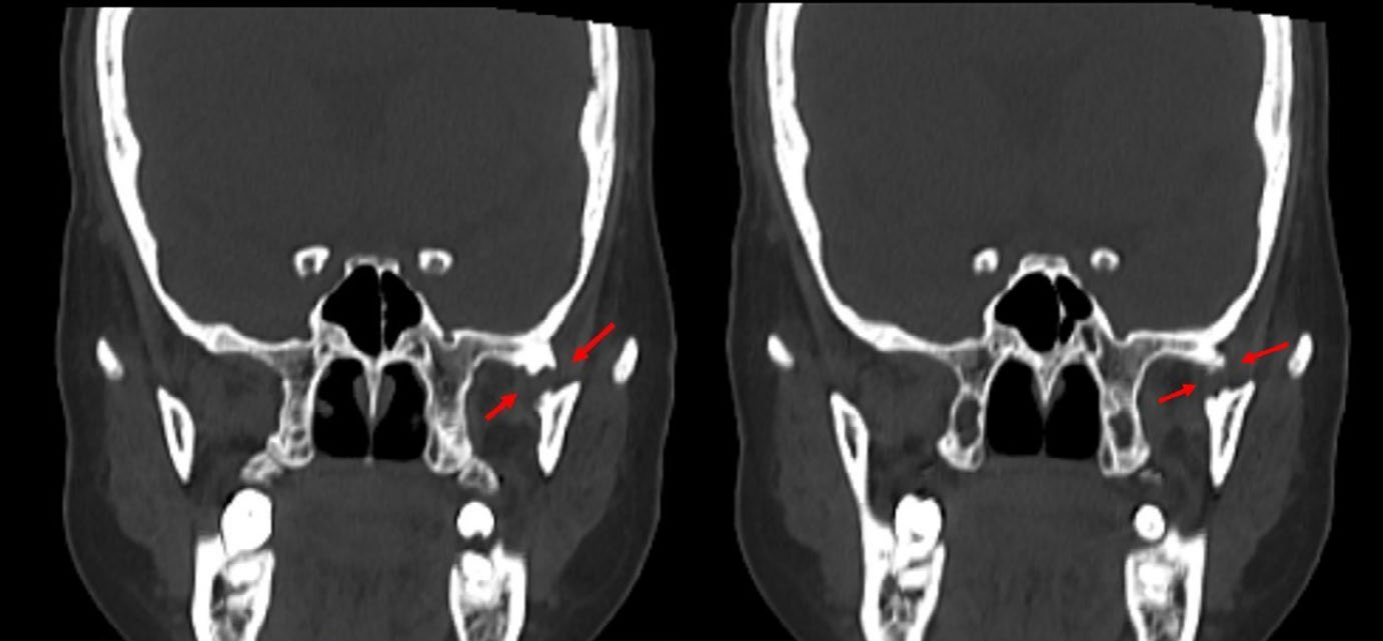
Coronal section of NCCT face. Arrow shows the bony bridge with intervening radiolucent band between the greater wing of sphenoid bone and the medial surface of ramus on the left side. (NCCT—non‐contrast computed tomography)

Orthopantomogram revealed flattening and notching of condylar head on the left side (Figure 10). NCCT (non‐contrast computed tomography)/MRI (magnetic resonance imaging) did not reveal any evidence of bony/ fibrous TMJ articular ankylotic changes. However, ossified bony bridge was visualized on the left side from the infratemporal fossa to the medial surface of the ramus with intervening radiolucency suggestive of extraarticular ankylosis (Figure 11).

## 5 DIAGNOSIS AND TREATMENT PLAN

Correlating the history and clinical findings overlapped on the radiological picture of extraarticular ankylosis, the patient was diagnosed of having fibrodysplasia ossificans progressiva (FOP). The patient was counseled for palliative management and was taken up under general anesthesia for the extraction of teeth 38 and 45 through the buccal approach. Post‐operative period was uneventful. No worsening of her existing mouth opening was seen after a follow‐up of 1 year. The patient and her daughter were counseled for genetic testing, but both denied the same.

## 6 LITERATURE REVIEW

Systematic review of literature was conducted to review the characteristics of maxillofacial involvement and treatment strategies for the restricted mandibular movements (RMM) in patients with FOP. The study was exempted from Institutional ethical committee approval.

## 7 INCLUSION CRITERIA

Case reports, case studies, case series, retrospective studies, prospective studies, observational studies, randomized control trials, and non‐randomized control trials with specific information on the involvement of maxillofacial region and RMM were included in the review. Articles in English or with English translation were preferred. No publication date or publication status limits were applied.

## 8 EXCLUSION CRITERIA

Studies on FOP patients with no specific details on maxillofacial involvement or RMM were excluded.

## 9 LITERATURE SEARCH

**FIGURE 12:**
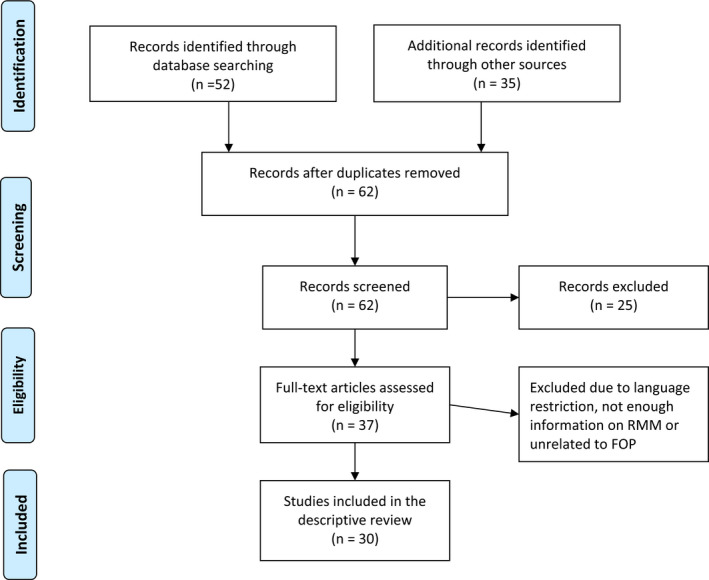
PRISMA flowchart (RMM—Restricted mandibular movements; FOP—Fibrodysplasia ossificans progressiva)

A systematic electronic search of PubMed, Medline‐Ovid, Springer Link, Embase, Scopus, Science Direct, and Cochrane Database was conducted in accordance with the Preferred Reporting Items for Systematic Reviews and Meta‐Analyses (PRISMA) statement on November 16, 2021 for articles in English (Figure 12). A manual search of the oral and maxillofacial surgery related journals including Journal of Oral and Maxillofacial Surgery, International Journal of Oral and Maxillofacial Surgery, British Journal of Oral and Maxillofacial Surgery, Journal of Craniomaxillofacial Surgery, Journal of Craniofacial Surgery, Journal of Maxillofacial Oral Surgery, Journal of Oral Surgery, Medicine and Pathology, Oral and Maxillofacial Surgery, Oral Surgery, Oral Medicine, Oral Pathology, Oral radiology, Plastic and Reconstructive Surgery and Journal of Craniomaxillofacial Trauma and Reconstruction. The search string used was ((Fibrodysplasia ossificans progressiva) OR (Stoneman syndrome) OR (Stoneman disease) OR (Munch Meyer disease) OR (Myositis ossificans progressive)) AND ((Temporomandibular Joint) OR (TMJ) OR (Jaw) OR (extraarticular)) AND ((Ankylosis) OR (Restriction)). The literature search was carried out by the first and the second authors separately and confirmed for uniformity.

## 10 RESULTS

62 articles were scanned after removing duplicates for relevance and availability of specific information related to restriction of mandibular movement (RMM) in FOP. 32 articles were excluded due to insufficient information, language other than English or due to the irrelevance to the topic. The 30 articles included in the review are presented in Table 1. Statistical analysis of the findings of the cases in the literature review was not possible due to the non‐uniformity of the reported findings. The review was confined to being a qualitative systematic review.

**TABLE 1:** Literature review of fibrodysplasia ossificans progressiva cases with restricted mandibular movements in literature.

S No	Author and year	Type of article	Sex	Age1*	Age2**	Contributory history for jaw involvement	Maxillofacial findings	Dental findings	Specific cause for reduced MIO	Treatment history	Other findings
1	Van Der Meij, 2006^6^	Case report	F	9	2 weeks before reporting	Fall from stairs 4 weeks earlier	MIO – 2 cm Deviation to Lt on MO	NA	Bony apposition on Medial side of Lt ZA	NA	Exostoses Lt clavicle after fracture during birth Exostosis medial side of Lt proximal tibia 3 years after a fracture Exostoses distal sides of bilateral humeri Bony apposition along SCM B/L Hallux deformity Shortened middle and distal phalanges of II to V toe Increased uptake along Lt ZA and Rt SCM in whole‐body scintigraphy
2	Aslan, 1999^4^	Case Report	F	NA	3 years before reporting	NA	Restricted jaw movement	Bad odor, Multiple carious teeth	B/L Bony TMJ Ankylosis	TMJ Arthroplasty could not establish intraoperative mouth opening but lead to HO between angle of the mandible and maxilla. HO excised during second operation led to complete re‐ankylosis 1 year post‐operatively	Neck, Rt arm, and thoracic movements restricted Ossified soft tissues neck, Rt arm, thoracic and pelvic areas
3	Young, 2007^11^	Case report	F	24	NA	Spontaneous and overnight restriction of mouth opening	MIO ‐ 0 ossification of buccinator/ masseter muscle	Multiple caries Gingivitis Impacted teeth	TMJ bony ankyloses Ossification of buccinator and masseter Enlarged coronoid process B/L	Extraction of molars under GA	‐Complete fusion of cervical spine and ribs Neck and trunk fused in an upright position Limitation of most joints except Lt knee and Lt ankle Rt arm ankylosed in flexed position Lt arm ankylosed in extended position Rt knee flexed,Lt leg extended Limited thoracic inspiratory excursion secondary to intercostal ankylosis
4	Mortazavi, 2012^13^	Case report	M	28	NA	NA	MIO – 5 mm Lateral jaw movements – 0 mm	Poor hygiene Multiple dental decays Dental abscess	HO trapezius, complete fusion of spine	Recurrent surgery for removal of a mass from scapula region at 3 years age and 2 months later leading to limitation of neck movement Dental abscess drainage and antibiotic therapy	Limitation of movement of knees, jaws, spine, shoulders, hips, and distortion of neck, incomplete extension of elbow, shortened first phalanx of thumb, Paraspinal muscle calcification
5	Wadenya, 2010^10^	Case report	M	20	9	Hit in the face with a baseball	Near complete mouth opening restriction	Multiple carious teeth	Complete fusion of condylar head to temporal bone	Biopsy for a facial swelling at 9 years Investigative TMJ surgery at 10 years Subcondylar osteotomy which established appropriate mouth opening for multiple restorations and extractions Tracheostomy for airway distress post‐surgery	DM type I, hypothyroidism, immobilization of every joint at 30 years, 90 degrees bent at waist, neck fixed to the Lt, Cane assisted ambulation
6	Herford, 2003^15^	Case report	M	24	14	NA	Complete mandibular restriction	Rampant decay and multiple abscessed teeth	Fusion of hypertrophic coronoid process with medial surface of zygoma	Intraoral coronoid gap arthroplasty with BFP interposition gradual reduction in mouth opening post‐surgery and maintained at 15 mm and 12 months post‐op	Multiple affected muscles and joints
7	Chichareon, 1999^14^	Case report	M	3	NA	NA	MIO <2 cm on presentation		Calcified mass of 1 X 2 cm at lower border of Rt Mandible diagnosed histologically as osteochondroma Developed a hard swelling of Rt Zygomatic complex and HO HO anterior to masseter connecting maxilla and mandible after I surgery Bony hard spicule on the lingual side of mandible and firm subcutaneous connective tissue bands of the Rt neck Outcome worse than initial presentation	Torticollis on the Rt neck fibrous bank excised after birth **I surgery**‐ Removal of bony mass (osteochondroma) which led to a MIO of 1 mm and 2mm lateral excursion **II Surgery** ‐ Combined intraoral and extraoral approach to remove the calcified mass and Rt coronoid excision led to a MIO of 5 mm **III Surgery –** Intraoral resection of calcification at the tip of coronoid process and body of the mandible	Calcified columns on the Rt neck Limited neck extension B/L Hallux valgus Limited flexion Rt elbow Calcification of Rt SCM near Rt clavicular head
8	Kriegbaum, 2013^7^	Case report	M	26	23	NA	MIO – 12 mm on presentation MIO – 8 mm post management	Multiple carious lesions	Bony projection uniting lateral aspect of ascending mandibular ramus with ZA Post‐surgical course resulted in exaggerated bony union between zygoma and mandible	Under GA; submandibular incision to separate sigmoid notch and ZA. Repeat surgical procedures to remove HO with adjunctive medical management (Salazopyrine, methylprednisolone bisphosphonates, and interposition with Goretex membrane)	Mild Psoriasis Bechterews disease No other musculoskeletal lesions developed
9	Sendur, 2006^5^	Case report	F	20	NA	NA	MIO – 5 mm Trend to ankylosis MIO – 10 mm after conservative therapy	NA	Trend to ankylosis; unspecified	Superficial and deep heating; analgesic currents; range of motion and stretching exercises	Greater toe shorter than other Generalized movement restriction in all joints Cervical vertebrae synostosis Calcifications of anterior and posterior longitudinal ligaments of lumbosacral joints Exostosis beneath Rt scapula and Lt axillary region, Lt hip joint, along the Lt femoral region from the distal to proximal end and on Lt elbow
10	Fernandes, 2014^20^	Case report	F	26	NA	NA	NA	Multiple caries	NA	Conservative dental procedures, oral hygiene instructions, recurrent topical fluoride applications, endodontic and restorative treatments.	NA
11	Orhan, 2012^3^	Case report	F	20	NA	NA	Restricted mouth opening	Lt Mandibular II molar pathology	Bony extension of Lt coronoid with ankylosis to the skull base medial to the Lt ZA in the area of Lt Temporalis Bifid condyle appearance Degenerative and condylar flattening B/L	NA	Confirmed ACVR mutation Walking disorder HO hips, ankles, elbows, and posture problem HO Rt Mastoid region Tall narrow cervical vertebral bodies with large posterior elements
12	Peter Renton, 1982^12^	Case series	M	5	7 (on prospective review)	NA	Normal mandibular movements till 5 yrs	NA	Gross abnormality of condyles with B/L short broad neck, heads wide and flat and marked spur formation	NA	Short great toes and thumbs Ossification of thoracic and abdominal wall musculature Broad femoral necks Clinodactyly hypoplasia of thumb hypoplasia of cervical vertebral bodies and fusion of nuchal arches
			M	NA	NA	NA	NA	NA	Broad condyles flattened articular surfaces Posterior spur	NA	B/L hallux valgus ossified masses in muscles in the thoracic wall hypoplasia of cervical vertebral bodies congenital anomalies of thumb congenital fusion of great toe metatarsals and proximal phalanges Lump of neck extending to Lt scapular region and developing shoulder stiffness
13	Connor, 1982^17^	Retrospective survey‐based study 44 cases surveyed; 34 examined; 3 died; 7 not traced	14F 9 M	Current average – 33.6 (SD=13.9)	5–26 (17.6±6.76)	3/34 – H/o trauma 3/34 – H/o dental therapy	Many had complete inability to mouth opening; Reddening of overlying skin leads to inability to mandibular movements in a few days	NA	NA	1/34 underwent ectopic bone excision but recurred	79% big toes shortened with single phalanx 15% stiff big toes of normal length 6% reduction defect of all digits 59% short thumbs due to short I metacarpals 44% fifth finger incurving
14	Cramer, 1981^16^	Case report	F	3 months	3 mo	NA	3 mo ‐Cyst like lesion in lower lip, corner of the mouth and buccal mucosa with reduced MIO 15 mo – Biopsy revealed bone 8 years – bony mass leading to TMJ Ankylosis 9 years – lesion recurrence 13 years – Lt cheek rock hard; lacked soft tissue nasal involvement, nodule in scalp, base of big toes	Dental decay and abscess	Bony mass in the region of lip and cheek	Biopsy at 15 months Resection of bony mass in the region of cheek but recurred Partial mandibulectomy 4 months later – Rt TMJ resection Prostheses for esthetics maintained at 15mm MIO for next 2 years	Short 4th 5th metacarpals Hypoplastic distal phalanges Soft tissue density ulnar styloid and plantar surfaces
15	Debeney, 1998^18^	Case report Of familial involvement	F	21	15 days earlier	Trauma to Rt mandibular angle 3 months before	Painful swelling Lt angle of mandible restricted mandibular opening at 25 mm on presentation Inability to translation Pain in coronoid on palpation Almost complete MIO restriction post‐surgery		Diffuse radio‐opaque structures in sigmoid notch Isolated calcification along Rt LPM on presentation Rt LPM calcification and ongoing Lt MPM calcification	NA	5 generations and 7 individuals affected Absolute MIO loss in all involved patients 75% females 25% males affected only Maxillofacial region Average age of jaw involvement is 23 years
16	Roberts, 2011^19^	Case report	F	44	NA	Toothache posterior region both upper and lower	HO B/L TMJ and Masseter Mouth opening less than 1 mm	Tooth ache upper and lower posterior region	NA	Dental extraction carried out under GA	Bedridden HO in trunk, shoulders, hips great toe phalanges malformed, shortened, fixed with ulnar deviation
			M	44	NA	NA	Mouth opening – 3 mm	Poor oral hygiene Large carious lesion in lower III molar Generalized horizontal bone loss in posterior mandible Interproximal calculus spurs Generalized PDL widening	NA	Refused treatment	NA
			F	07	NA	NA	Hypoplastic mandible	8 carious deciduous teeth	No features of ankylosis	02 teeth restored by atraumatic restorative treatment 06 caries arrested Topical fluoride application	Irregular bone swellings on the back Restricted movement of neck and shoulders
			F	Died at 52 years; surveyed later	NA	NA	MIO – 12 mm	NA	NA	Oral hygiene, scaling. 2 incisors extracted for feeding	Frequent respiratory problems Fusion of cervical spine Chin stuck to the chest unable to walk or sit Died at 52 years, extraocular muscles, fingertips, and tongue movement only at death
			F	2	NA	NA	Only mandibular hypoplasia	NA	NA	NA	Limitation of arm movements Hallux valgus Shortening of great toes Firm swellings back and scalp damage to upper lip consequent to trauma
18	Carvalho, 2011^21^	Case report	M	13	7	Trauma to Rt cheek at 7 years	Extraarticular ankyloses Retrognathia Rt sided abnormal LPP Flat condyle Rt Lt sided ossification of SHL	NA	Pterygoid muscle ossification Rt side extending to mandible	NA	NA
			F	21	10	Mandibular nerve blocks and stretching during treatment	Extraarticular ankylosis Retrognathia B/L abnormal LPP; Large in Rt side Flat condyle Rt B/L ossification of SHL	NA	B/L Pterygoid muscle ossification Rt side extending to mandible	NA	NA
			M	22	9	Submandibular and posterior neck swelling resection	Extraarticular ankyloses Retrognathia B/L abnormal LPP; Large in Rt side Flat condyle Rt B/L ossification of SHL	NA	Pterygoid muscle ossification Rt side extending to mandible	NA	NA
19	Nunnelly, 1986^22^	Case series	F	40	5	Neck injury	Complete mandibular restriction Small nodular masses along margins of mandible extending to TMJ	NA	Well defined mass in Infratemporal fossa extending to coronoid HO extending inferiorly from occiput in the distribution of intraspinous ligaments	NA	‐All joints involved ‐ Rigid neck
			F	26	1	Posterior neck mass biopsy	Severe trismus with dysarthria Bony mass in Rt hypoglossal bone region	NA	Marked hypertrophy of LPP Ossification of LPM	NA	All joints involved; Rigid neck
			M	19	1	NA	Complete restriction of mandibular movement MIO at 1 mm Lateral movement at 6 mm Palpable bony mass along anterior border of Rt masseter muscle extending from ZA to Inferior border of mandible B/L bony mass in temporal and suprahyoid musculature	NA	Bone continuous from Zygoma in direct apposition to lateral aspect of body of the mandible	NA	Neck fixed to Rt
20	Mori, 2000^23^	Case report	M	18	NA	NA	MIO – 5 mm	16 mm overjet 5mm overbite ‐Abnormal eruption pattern and positioning ‐Rt upper II molar in scissors bite with caries ‐ B/L Lower II molar distal tipping ‐ B/L lower III molars horizontal ‐B/L Upper III molar poor hygiene	B/L condylar head flattening shortening of condylar process Hypertrophy of Rt coronoid process	Hallux valgus corrected at 1 year	Hallux valgus Multiple joint contractures Marked spinal deformation Restrictive lung disease with 40% Vital capacity Wolff‐Parkinson‐White syndrome Mild aortic dilation
21	Duan, 2010^24^	Case report	M	17	4	NA	Asymmetric face MIO – 1 mm Jugomaxillary muscle effective when bite down	Regular dentition	HO Rt Pterygoid process and mandible	Exploratory operation o Rt hip joint and excision of osteophyma Removal of HO via intraoral approach MIO‐1 cm at 2 years follow‐up	Solid fixed subcutaneous nodules in back with slightly limited movement of neck and lower back Rigid B/L hip Solid nodules Rt wrist Ankylosis Rt hip Inability to bend at waist Claudication aggravated during walking Loss of cervical physiologic curvature Non‐scoliosis soine 2 typical bony intumescences at T12 L2 Limited movement of Rt hip Increased Alkaline phosphatase Anomaly of centrum vertebrae
22	Leavitt, 2009^25^	Case report	M	50	At birth	NA	MIO – 3 mm Firm, swollen tender swelling Lt inferior border of mandible till the level of thyroid cartilage	Impacted III molars Increased PDL with II molars	Bony extension of Rt coronoid process to skull base Lt coronoid process lengthened Rt condylar head irregular borders HO stylohyoid ligament Transient Liquefaction of Lt mylohyoid muscle	NA	Wheel chair bound
23	Geddis‐Regan, 2018^26^	Case report	M	45	NA	Local anesthesia administration as a child	MIO – 2 mm	Gross carious teeth	Slight narrowing of joint space Small osteophytes on Rt condylar head B/L condylar flattening	Restoration of posterior teeth with chronic apical disease in both left and right mandibular teeth	Chronic ossification of intercostal spaces
24	Crofford, 1990^27^	Case series	M	NA	NA	NA	MIO ‐ Nil	NA	Solid ossified bridge Lt side anterior ZA to Lt coronoid process	Resection of bony mass MIO maintained at 1 mm after 8 months	B/L hallux valgus at birth Numerous swellings with minor trauma stiff gait Calcified lesions of the wrist Paravertebral scapular shoulder girdle HO Foreshortening and fusion of phalanges Lt axilla HO
			M	NA	15.5	NA	MIO – 5 mm	NA	Ectopic ossification arising from medial surface of Rt mandibular ramus and ending at Rt Pterygoid plate coinciding with MPM Increased Technetium 99 uptake Rt ramus of mandible extending to soft tissues of cheek	Removal of osseous tether and successful muscle resection with a 4 mm MIO at 2 months.	Hypospadias Ambiguous genitalia at birth 46XY karyotyping Calcification of Rt paravertebral regions Dextroscoliosis of thoracic and lumbar regions B/L hallux valgus Shortening of great toes rudimentary proximal phalanx exostoses medial aspect of distal femurs and proximal tibia B/L HO soft tissues neck and thoracolumbar spine Bony columns in axilla and soft tissues around Rt proximal femur Reduced range of motion of neck, back and shoulders
25	Okuno, 2017^28^	Case series	F	29	13	NA	MIO ‐ 5 mm		HO mentum and hyoid bone in the region of geniohyoid, mylohyoid and anterior belly of digastric	Pulpectomy Shaving of upper and lower incagainst a table and losing 02isors to establish mouth opening for feeding	B/L hallux valgus HO posterior neck Malpositioned, unbalanced and marked scoliosis
			M	39	02	Trauma after bumping against a table and losing 02 incisors	MIO−2 mm	Multiple decayed teeth	HO from mentum to hyoid bone Hypertrophy of Lt coronoid process		Torticollis Spinal ankyloses
			F	62	39	‐	MIO−10 mm	‐	HO Medial Pterygoid muscle B/L	‐	Multiple nodules upper limbs, lower limbs. Spinal column ankylosis HO Neck muscles
26	Susami, 2012^29^	Case report	M	8	NA	NA	Developing counterclockwise rotation of mandible during growth Class II Skeletal malocclusion with Maxillary Prognathism	MIO – 5 mm Class II molar relationship with increased overjet and overbite Impacted III molars	Deformed B/L Condylar heads Widening of Rt coronoid process HO anterior edge of coronoid process	Surgery of Hallux Valgus 07 molar extraction	Hallux Valgus HO vertebral column Scoliosis head tilt
27	Pachajoa, 2015^30^	Case series	M	17	NA	NA	Class II Malocclusion Facial asymmetry Micrognathia Sparse eyebrows MIO‐3 mm	Dental hyperpigmentation Spaced inferior teeth retrognathism dysphonia	alteration of mandibular condyles with arthrosis Coronoid hyperplasia		Unclear family history Thoracolumbar scoliosis Of Lt convexity with vertex at T11‐T12 2^nd^ curvature with Rt convexity with vertex at L5 Rt pelvic tilt Verticalization of acetabulae Lateralization of Rt femoral head secondary to subluxation Severe restrictive lung defect on spirometry Moderate mental retardation Limitation of nek movements B/L thumb hypoplasia Generalized atrophy of muscles of hands Arachnodactyly Upward tilt of rt hemipelvis 30‐degree fixed flexion of hip B/L hypoplasia of I metatarsal B/L ulnar deviation of halluces
			M	11	NA	NA	Skeletal Class II Malocclusion Facial asymmetry Micrognathia Sparse eyebrows MIO – 4 mm	NA	NA	NA	HO scapular regions with humeral diaphysis HO nuchal region Tricuspid insufficiency Lt thoracic hump Hypoplasia of I metacarpal and short phalanges Muscular atrophy and hypertonicity of muscles on Rt hemibody Ankylosis of Rt glenohumeral joint B/L hypoplasia of I metatarsal
28	Braga, 2011^31^	Case series	F	23	NA	NA	MIO‐ 3 mm	Anterior open bite	No evidence of frank ankylosis	Mouth opening exercises resulted at MIO 17 mm	Walking difficulty B/L congenital malformation of big toes Stiffness distributed globally Lack of skeletal mobility Reduced peripheral joint mobility Thoracic scoliosis, severe lumbar lordosis HO paravertebral muscle Decreased expansiveness of chest stiffness of hips and shoulders significant muscular atrophy B/L valgus knee I toes of both feet were significantly shorter than others Diffuse ossification of soft tissues of chest
29	Vashisht, 2006^32^	Case report	F	NA	12	Swelling of lower jaw Lt side with toothache 10 days before reporting. Submandibular induration and sublingual swelling with inability to protrude tongue	MIO less than 1 cm	NA	NA	Dental extraction under LA at 5 years age Further dental extraction and submandibular drain	B/L hallux valgus No neck movements B/L shoulders and upper chest involvement Scoliosis Restrictive lung function Knee restriction Left ventricular hypertrophy Tricuspid regurgitation
30	Sellami, 2015^33^	Case report	F	24	NA	NA	MIO−15 mm	NA	HO Rt Pterygoid muscles	NA	Congenital B/L malformed toes with valgus formation Lt sided indurated mass in the SCM with torticollis

Note

Abbreviations: B/L, Bilateral; F, Female; GA, General anesthesia; HO, Heterotopic ossification; LA, Local anesthesia; LPM, Lateral pterygoid muscle; LPP, Lateral pterygoid process; Lt, Left; M, Male; MIO, Maximal interincisal opening; MO, Mouth opening; MPM, Medial pterygoid muscle; NA, Not applicable; PDL, Periodontal ligament; Rt, Right; SCM, Sternocleidomastoid; SHL, Stylohyoid ligament; TMJ, Temporomandibular Joint; ZA, Zygomatic arch.

The following are the important observations from the literature review: In patients with FOP, the age of onset of RMM is variable, some manifesting even congenitally. However, most of the patients manifest RMM during the second decade of their life. There is a very slight female preponderance of FOP patients with RMM (30 males and 34 females). Most of the patients have been pre‐diagnosed with FOP when they report with RMM, but delayed diagnosis or previous misdiagnosis is a common feature. The onset of RMM may be spontaneous or post‐trauma (including iatrogenic causes like surgery/biopsy in previously undiagnosed patients, nerve blocks, or jaw stretching during dental therapy). The most common dental manifestation includes multiple decayed teeth with or without abscess formation. The most common cause of RMM in FOP is extraarticular ankylosis but classic TMJ Bony ankylosis is also evident in some cases. Extraarticular ankylosis also present with condylar flattening and tendency to bifid condyle on radiographic examination. Mandibular retrognathia has been reported in some cases but the same fails to be mentioned in most of the reported cases possibly due to the later onset of TMJA. Great toe malformation (GTM) is found in a large majority of patients, but the phenotypical expression is variable and therefore is not pathognomonic of the disease. FOP patients with RMM always had other joint involvements with restricted movements except in a familial cluster of patients with exclusive maxillofacial involvement. Majority of the patients were deferred surgical management while in those patients in whom surgery was attempted, recurrence of TMJA was inevitable, fast, and more debilitating. Most of the patients underwent palliative treatment with dental extractions, restorations, or endodontic treatment under general anesthesia (GA). Preventive measures like topical fluoride application, oral hygiene instructions, and oral prophylaxis have also been instituted.

## 11 DISCUSSION

Temporomandibular joint ankyloses is a debilitating condition, the diagnosis of which is a prompt indication for absolute surgical management. FOP is a recognized cause of TMJA, but the awareness of the condition appears to be low among clinicians due to its rarity. Studies reveal that 90% patients of FOP have a history of misdiagnosis and 67% undergo unwarranted diagnostic procedures resulting in flare ups.^3^ Our patient was not diagnosed of FOP but was being managed for osteoarthritis for 25 years. The exact events leading to the diagnosis of FOP in the cases presented in the review are not exactly known but instances of misdiagnosis and delayed diagnosis exist.

FOP is an extremely rare catastrophic genetic disorder affecting 1 in 1.7 million^4^ (Range: 0.6 to 2 per million) with a current approximate estimate of 3900 cases worldwide. It may occur sporadically or inherited in an autosomal dominant (AD) pattern with variable expressivity but complete penetrance.^5^ The rarity of the disease precludes large single‐center cohorts for review. However, existence of worldwide associations like International FOP association(IFOPA) has enabled availability of larger cohorts for review.

FOP results in heterotopic ossification(HO) of ligaments, tendons, and skeletal muscles^6^ progressing similar to embryonic skeletal formation^6^ leading to ectopic skeletenogenesis.^7^ The etiology is controversial but overexpression of BMP‐4, by an altered inhibitory mechanism (altered NOG polypeptide coded by NOG; NOG deactivates BMP 4), is the most accepted mechanism and the locus has been mapped to 17q21‐22.^8^ ACVR1 gene mutation has also been found in a majority of patients with FOP.^9^ Gene analysis for confirmation may not be available or possible in all patients due to consent and affordability reasons.

The average age of onset of ossification is 5 years.^5^ Trauma is the most common trigger, although spontaneous onset is also reported.^6^ Unresolving erythematous painful nodules occur in the subcutaneous and muscular tissues that lead to progressive ossification.^10^ The diagnostic triad for this disorder is GTM, usually microdactyly of great toes, progressive HO in an endochondral manner, and HO in characteristic anatomical patterns progressing from cranial, proximal, axial, and dorsal regions to caudal, distal, appendicular, and ventral regions.^11^ Extraocular muscles, diaphragm, muscles of deglutition, heart, tongue, abdominal wall, perineum, and viscera are spared.^11^, ^12^ However, GTM is variable, occurs in only 75–95% of the cases, and therefore is not pathognomonic of the disease.^5^, ^12^ Biochemical investigations are generally normal but discrete increase in erythrocyte sedimentation rate^13^ and alkaline phosphatase^14^ has been reported during flare‐up episodes.

Diagnosis is mainly clinical and radiological, although genetic linkage analysis is confirmatory.^15^ Ultrasonography and magnetic resonance imaging may be useful in confirming early cases due to their ability to detect edema and neovascularization. Bone scintigraphy helps detecting active areas of bone formation.^14^ Non‐contrast computed tomography will help identify the anatomical extent of the bone formation.^3^ However, in clinically well‐established cases, conventional whole‐body radiographs are sufficient for diagnosis.^6^, ^16^ The phenotypic expression and longevity are variable in this condition depending on the areas affected. Death if occurs due to FOP is generally due to spine and rib cage ankylosis.^6^

The most debilitating manifestation of FOP is the ossification of masticatory muscles TMJA.^3^ TMJ is one of the last joints to be involved,^3^, ^16^ but 71% of the patients with FOP are affected with TMJA^15^ and 68% of them have been found to be extraarticular in nature.^17^ Restriction of mandibular opening occurs at an average age of 18 years and that the average age with no jaw involvement is 12.1 years.^17^ On analysis of literature, release of ankylosis have always resulted in short‐lived improvement and a guaranteed recurrence with exaggerated bone formation.^6^ Several treatment options have been explored but no effective treatment exists for this disorder till date.^13^

The reasons for presenting this fairly straight forward case report with review of literature are manifold: Firstly, FOP is rare but nevertheless an important cause of TMJA and has to be considered in every patient presenting with TMJA. Some patients may manifest isolated TMJA prior to developing other lesions and the maxillofacial surgeon/dental surgeon happens to be the first consultant. Though not confirmatory, examination of the entire skeletal system especially GTM, hallux valgus should be eliminated in these patients. Absence of GTM does not eliminate the possibility of FOP as seen in our case but its presence mandates further evaluation. Secondly, considering the literature, TMJA in FOP happens to be an absolute contraindication for surgery. Any surgery for release of ankylosis will lead to recurrence and worsening of the condition.^18^ Perhaps, surgery is not the solution to all TMJA. Thirdly, identification of this condition is important to institute preventive dentistry to prevent secondary manifestations of TMJA.^6^ Fourthly, routine dental treatment, for example, inferior alveolar nerve block (IANB) may precipitate HO in FOP patients.^19^ Therefore, GTM should be a part of examination of normal young patients to prevent iatrogenic precipitation of this condition due to routine oral procedures. Fifthly, many patients may report with dental complications of long‐standing TMJA in FOP. In such patients, any intramuscular administration of local anesthesia, for example, IANB is contraindicated for reasons of intramuscular HO.^20^ Such cases must be treated under GA administered with a fiberoptic bronchoscope. Overzealous manipulation of the neck and overstretching of the jaw should be avoided.^11^ Dental extractions should be carried as atraumatic as possible through the buccal approach.^10^, ^11^ Sixthly, the lifespan of these patients seems to be increasing, and the individuals are fertile.^16^ Known AD pattern of inheritance makes prompt diagnosis and genetic counseling mandatory.^18^ Lastly, the awareness of the existence of this disorder is lacking among clinicians leading to unnecessary invasive diagnostic procedures (like biopsy) leading to worsening of the condition.^20^ Especially, in developing countries like India, the data on the patients with FOP are lacking but the patients with TMJA are high. In short, awareness of FOP among the maxillofacial fraternity will prevent precipitation in susceptible normal patients, exacerbation in the existing ones, and consideration palliative procedures for dental ailments in patients with FOP.

## CONFLICTS OF INTEREST

None declared.

## AUTHOR CONTRIBUTIONS

Kavish Kapoor was a major contributor in working up the case and arriving at the final diagnosis. Arunkumar Shadamarshan Rengasayee contributed by providing the clinical material and working up the case for management; involved in revising the manuscript critically for important intellectual content; agreed to be accountable for all aspects of the work in ensuring that questions related to the accuracy or integrity of any part of the work are appropriately investigated and resolved. Rohit Sharma contributed in part by working up the case and arriving at the final diagnosis and gave the final approval of the version to be published. Nitesh Agrawal contributed to drafting the manuscript, appropriate literature review and interpretation to be included in the manuscript.

## ETHICAL APPROVAL

Necessary ethics committee approval and informed patient consent have been obtained for the case study. The systematic review has been exempted from ethical committee approval.

## CONSENT

Informed patient consent has been duly obtained during the procedure and for the publication of the photographs.

## Data Availability

The data that support the findings of this study are available on request from the corresponding author. The data are not publicly available due to privacy or ethical restrictions.
